# Evaluating perfusion and metabolic responses of microvascular free flaps to ischemia, reperfusion and fluid resuscitation during septic shock in a large animal model

**DOI:** 10.1038/s41684-026-01747-0

**Published:** 2026-05-20

**Authors:** Daniel Stephan, Alexander Ziebart, Robert Ruemmler, Miriam Renz, Bilal Al-Nawas, Sebastian Blatt, Peer. W. Kämmerer, Daniel G. E. Thiem

**Affiliations:** 1 Department of Oral and Maxillofacial Surgery, Facial Plastic Surgery, University Medical Centre of the Johannes Gutenberg-University Mainz, Mainz, Germany; 2 Department of Anaesthesiology, University Medical Centre of the Johannes Gutenberg-University Mainz, Mainz, Germany

**Keywords:** Experimental models of disease, Reconstruction

## Abstract

Microvascular free flap transfer is an essential procedure in head and neck reconstructive surgery, with total flap loss representing the primary complication. Sepsis has been identified as a critical risk factor contributing to flap failure. Here we aimed to evaluate the impact of septic shock on free flap vitality using a large animal model and further investigated the flap-specific effects of different fluid resuscitation strategies on flap viability and metabolic response. A prospective, randomized trial was conducted on 31 juvenile male pigs to assess the effects of 3 fluid resuscitation strategies following septic shock induced by lipopolysaccharide infusion. The strategies included a balanced crystalloid solution, a colloid, and a combination of crystalloid and resveratrol. Free flap vitality was continuously monitored using hyperspectral imaging and blood gas analysis, comparing intraflap venous blood with central venous blood. The crystalloid group most effectively maintained higher oxygen saturation and lower lactate levels. Conversely, the colloid group exhibited lower tissue oxygen saturation and higher lactate levels. The combination of crystalloid and resveratrol stabilized the hemoglobin and tissue water indices. The study demonstrated a significant influence of different fluid resuscitation strategies on metabolic and perfusion outcomes of free flaps under septic conditions. Administration of crystalloid suggests optimized oxygen delivery and utilization, whereas the combination with resveratrol provided an improved balance of blood volume and hydration, hence preventing fluid overload.

## Abstract (web summary)

Using a pig model, the authors evaluated the impact of septic shock on free flap vitality and further investigated the effects of different fluid resuscitation strategies on flap viability and metabolic responses.

## Main

Microvascular free flap transfer is an essential technique in head and neck surgery, often used to reconstruct severe tissue defects resulting from tumor resection or traumatic injuries. This method represents the gold standard for restoring both functional capabilities and aesthetic appearance^1^. The overall success rate of free flaps typically exceeds 93%, mainly due to the high success rate of simpler fasciocutaneous flaps such as the radial forearm flap. The reliability is underscored by a recent study reporting a total flap loss rate of 2.1% in a review of 44,031 free flaps^2^. However, challenges persist, particularly with more complex perforator or osteocutaneous flaps, which are associated with more variable outcomes and higher complication rates^1,3,4^.

The primary challenge in microvascular tissue transfer remains the risk of total flap loss due to impaired perfusion, a substantial complication leading to increased morbidity^5^. Malperfusion due to vascular compromise, including arterial or venal occlusion, represents the main reason for flap failure, emphasizing the urgency of surgical revision^2^. The success rate of flap rescue diminishes over time, with salvage operations conducted within the first 24 h showing a success rate of 93.8%. By contrast, only 12.1% of flap revisions performed on postoperative day 3 were reported to be successful^2^. Early detection of impaired flap perfusion therefore remains critical for flap survival.

Beyond vascular compromise, postoperative infections leading to sepsis also contribute to late flap failures. Sepsis, defined by critical organ dysfunction triggered by a dysregulated immune response, can escalate to septic shock if accompanied by hypotension requiring vasopressors^6^. The postoperative period following extensive reconstructive surgeries is particularly vulnerable to sepsis owing to factors such as surgical invasiveness, patient health status and the nature of postoperative care^7^. In a recent study of 24,257 patients who underwent flap reconstruction, 511 developed postoperative sepsis, representing an incidence rate of 2.1% (ref. ^7^). These patients were more likely to experience severe complications, including septic shock, increased risk of 30-day mortality and total flap loss. Although less frequent than vascular occlusion, sepsis has been identified as a primary reason for late flap failure occurring after the seventh postoperative day^8^, highlighting the importance of identifying and avoiding postoperative infections to enhance patient outcome and flap vitality.

Considering the overall incidence of sepsis following reconstructive flap surgery, which ranges from 0.5% to 3.7%, comparable to other serious complications such as deep venous thrombosis (1.3%) and partial flap loss (2.2%), the risks associated with sepsis must be thoroughly addressed^7,8^. Recent studies further highlight the influence of underlying conditions such as metabolic syndrome, which substantially increases the risk of sepsis and septic shock following head and neck microvascular reconstruction^9^. However, available data on metabolic changes and flap vitality during sepsis are still very limited, particularly regarding flap-specific alterations.

Due to the limitations of retrospective data analysis and ethical constraints associated with conducting controlled human trials, the use of large animal models is critical for advancing our understanding of therapeutic interventions. Pigs (*Sus scrofa*), in particular, are highly valuable due to their anatomical and physiological similarities to humans. These similarities make them an ideal model for studying complex conditions such as sepsis in the context of flap vitality and survival^10,11^. Consequently, this study investigates the impact of sepsis and various therapeutic approaches on free flap vitality using a large animal model previously established and validated by our group^12,13^. In addition, this model provides broader insight into free flap physiology under ischemia, reperfusion and systemic inflammatory stress, offering a comprehensive view of perfusion and metabolic dynamics.

## Results

This study explores the effectiveness of three distinct fluid resuscitation strategies on the vitality of free flaps in a septic shock model induced by lipopolysaccharide (LPS) infusion. The primary aim was to evaluate the impact of surgical intervention and septic shock on flap vitality without differentiation among experimental groups. Upon the induction of shock, animals were randomized into three groups to receive either a balanced crystalloid solution (Sterofundin), a colloid (Gelafundin) or a combination of crystalloid and resveratrol (Sterofundin + resveratrol). The vitality of the flaps was assessed using hyperspectral imaging (HSI) and blood gas analysis of intraflap venous blood, complemented by systemic evaluations using central venous blood gas analysis and HSI of regular abdominal skin, which served as a control.

### HSI (absolute values)

**Fig. 1:**
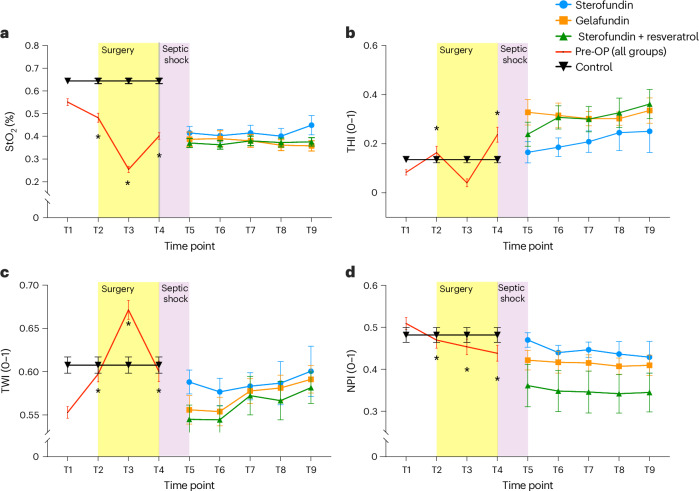
HSI (absolute values). **a**–**d**, StO_2_ (**a**), THI (**b**), TWI (**c**) and NPI (**d**) are plotted as line charts across multiple measurement time points from T1 to T9, with T3 immediately before and T4 immediately after flap reperfusion. The yellow area indicates the surgical procedure comparing free flap tissue with abdominal skin as a control. For the control tissue, values displayed from T1 to T4 originate from a single baseline measurement (T1); the points shown at T2–T4 were duplicated solely for graphical continuity, were not newly acquired and were therefore excluded from statistical analysis. Pre-OP refers to preoperative measurements. Pretherapeutic values represent aggregated data from all animals before group allocation. The violet area reflects the shock phase with subsequent assessment of 3 different treatment groups comparing a balanced crystalloid solution (Sterofundin, *n* = 10), a colloid (Gelafundin, *n* = 11), and a combination of crystalloid and resveratrol (Sterofundin + resveratrol, *n* = 10). Data represent mean ± standard error of the mean. Statistical analysis during surgery (T1–T4) was performed using multiple paired *t*-tests versus baseline (T1); post-shock analysis (T5–T9) was performed using a mixed-effects model fit with restricted maximum likelihood. Statistical significance was set at *P < 0.05.

During surgery, no differences between the groups were observed (tissue oxygen saturation (StO_2_): *P* = 0.827, *F*(2, 28) = 0.191; total hemoglobin index (THI): *P* = 0.167, *F*(2, 28) = 1.913; tissue water index (TWI): *P* = 0.604, *F*(2, 28) = 0.513; near-infrared perfusion index (NPI): *P* = 0.092, *F*(2, 28) = 2.600); therefore, all 3 groups are displayed as one (red line) (Fig. 1).

As shown in Fig. 1, HSI analysis of the flap during surgery indicated a significant decrease in StO_2_ during the surgery compared with baseline values (T1). This was followed by a significant increase after successful reanastomosis and reperfusion, as revealed by multiple *t*-tests (Fig. 1a, T1 versus T2: *P* = 0.005, *t* = 3.040, d.f. 30; T1 versus T3: *P* < 0.001, *t* = 17.59, d.f. 29; T1 versus T4: *P* < 0.001, *t* = 7.681, d.f. 29; T2 versus T3: *P* < 0.001, *t* = 7.840, d.f. 29; T2 versus T4: *P* < 0.001, *t* = 5.395, d.f. 29; T3 versus T4: *P* < 0.001, *t* = 5.859, d.f. 28). Similarly, THI decreased during surgery, which transitioned into an exaggerated recompensation and subsequent increase after reperfusion (Fig. 1b, T1 versus T2: *P* < 0.001, *t* = 3.710, d.f. 30; T1 versus T3: *P* = 0.062, *t* = 1.945, d.f. 29, T1 versus T4: *P* < 0.001, *t* = 5.469, d.f. 29). On the contrary, TWI increased throughout the surgical procedure up to T3, with a rapid decrease immediately after reperfusion (Fig. 1c, T1 versus T2: *P* < 0.001, *t* = 7.629, d.f. 30; T1 versus T3: *P* < 0.001, *t* = 9.651, d.f. 29, T1 versus T4: *P* < 0.001, *t* = 5.437, d.f. 29). As shown in Fig. 1d, the NPI continuously decreased during surgery (T1 versus T2: *P* = 0.001, *t* = 3.633, d.f. 30; T1 versus T3: *P* < 0.001, *t* = 3.775, d.f. 29, T1 versus T4: *P* < 0.001, *t* = 6.842, d.f. 29). Notably, for the control group, only one initial baseline HSI value of the abdominal skin was recorded, given the absence of further procedures causing systemic effects during the surgery. Therefore, the control values were expected to remain stable and were duplicated across all time points (T1–T4) for graphical visualization. However, no statistical analysis was performed with duplicated values.

Post-shock analysis using a mixed-effects model fit with the restricted maximum likelihood method revealed no significant differences in StO_2_ (*P* = 0.403, *F*(2, 28) = 0.938), THI (*P* = 0.181, *F*(2, 28) = 1.819), TWI (*P* = 0.576, *F*(2, 28) = 0.563) or NPI (*P* = 0.131, *F*(2, 28) = 2.191) between the 3 therapeutic groups upon induction of septic shock.

### HSI (relative values)

**Fig. 2:**
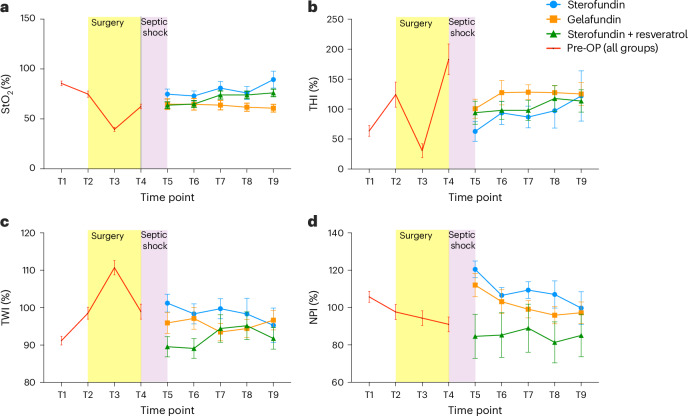
HSI (relative values). **a**–**d**, StO_2_ (**a**), THI (**b**), TWI (**c**) and NPI (**d**) relative to control (abdominal skin) values are plotted as line charts across multiple measurement time points from T1 to T9, with T3 immediately before and T4 immediately after flap reperfusion. The yellow area indicates the surgical procedure comparing free flap tissue and abdominal skin as a control. Pretherapeutic values represent aggregated data from all animals before group allocation. The violet area reflects the shock phase with subsequent assessment of 3 different treatment groups comparing a balanced crystalloid solution (Sterofundin, *n* = 10), a colloid (Gelafundin, *n* = 11), and a combination of crystalloid and resveratrol (Sterofundin + resveratrol, *n* = 10). Data represent mean ± standard error of the mean. Statistical analysis during surgery (T1–T4) and post-shock (T5–T9) was performed using a mixed-effects model fit with restricted maximum likelihood. Statistical significance was set at *P < 0.05. No individual post-hoc significant time points are indicated in this figure.

Considering the systemic impact of septic shock and fluid resuscitation therapy, HSI results for each time point were normalized against mean control values (set at 100%) from the abdominal skin at each specific time point. HSI changes during surgery are consistent with the previous results as depicted in Fig. 1. Post-shock analysis using a mixed-effects model revealed a significant difference in StO_2_ across the 3 therapeutic groups (Fig. 2a, *P* = 0.046, *F*(2, 28) = 3.442). The crystalloid group exhibited the highest StO_2_ levels in the free flap, whereas the lowest levels were observed in the colloid group, with these differences becoming increasingly pronounced over time. Although no significant influence of the therapy was detected regarding THI (Fig. 2b, *P* = 0.45, *F*(2, 28) = 0.822), the colloid group tended to display the highest THI values. By contrast, as presented in Fig. 2c,d, the combination of time and therapy exerted a significant impact on TWI (*P* < 0.001, *F*(8, 106) = 3.795) and NPI (*P* = 0.001, *F*(8, 106) = 3.519), as revealed by mixed-effects model. Immediately following circulatory stabilization (T6), the highest TWI and NPI values were recorded in the crystalloid group, while the lowest were seen in the resveratrol group. Over time, values across all groups gradually converged, with similar levels achieved at T9.

### HSI, individual therapy groups (relative values)

Given the significant influence of therapeutic strategies on free flap perfusion, each group was compared with its corresponding control group (abdominal skin) to evaluate the difference between flap-specific and systemic response.

**Fig. 3:**
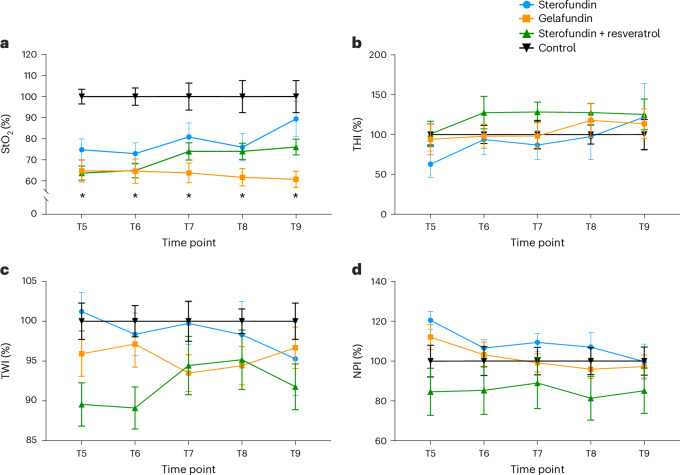
HSI, individual therapy groups (relative values). **a**–**d**, StO_2_ (**a**), THI (**b**), TWI (**c**) and NPI (**d**) relative to control values during the post-shock phase are plotted as line charts across multiple measurement time points from T5 to T9. HSI values from the free flap of the 3 different treatment groups including a balanced crystalloid solution (Sterofundin, *n* = 10), a colloid (Gelafundin, *n* = 11) and a combination of crystalloid and resveratrol (Sterofundin + resveratrol, *n* = 10) are compared with their corresponding control (abdominal skin), which was normalized to 100%. Data represent mean ± standard error of the mean. Statistical analysis was performed using a mixed-effects model fit with restricted maximum likelihood, with post-hoc comparisons adjusted using Šidák’s correction. Statistical significance was set at *P < 0.05. Asterisks indicate individual time points with significant post-hoc differences in **a** for the Sterofundin group.

Mixed-effects model revealed significantly lower oxygen saturation levels (StO_2_) of the free flap compared with abdominal skin in all experimental groups (Fig. 3a; crystalloid: *P* < 0.001, *F*(1, 18) = 14.09; colloid: *P* < 0.001, *F*(1, 20) = 37.00; resveratrol: *P* < 0,001, *F*(1, 17) = 44.42). Post-hoc comparisons with Šidák’s correction identified significant pairwise differences at all analyzed time points in Fig. 3a. During the observation period, StO_2_ levels of the crystalloid group gradually increased in the flap, reaching levels comparable to the control. Only a modest increase was observed in the resveratrol group remaining lower than control values throughout the whole experiment. By contrast, the colloid group exhibited a slight decline in StO_2_ over time. Mixed-effects model analysis revealed no difference between the 3 therapeutic groups regarding THI (Fig. 3b; crystalloid: *P* = 0.734, *F*(1, 18) = 0.119; colloid: *P* = 0.173, *F*(1, 20) = 1.998; resveratrol: *P* = 0.857, *F*(1, 17) = 0.033). The crystalloid group showed the highest TWI values in the free flap compared with control, although the differences were not significant. Figure 3c further depicts no difference in TWI between the free flap and abdominal skin (control) for any of the three experimental groups. Although both the colloid and resveratrol groups showed a tendency towards lower TWI levels in the free flap, these differences were not statistically significant (crystalloid: *P* = 0.625, *F*(1, 18) = 0.248; colloid: *P* = 0.262, *F*(1, 20) = 1.330; resveratrol: *P* = 0.056, *F*(1, 17) = 4.213). Whereas a significant difference between the free flap and abdominal skin was found in the colloid group for the interaction of time and therapy, with a higher NPI immediately after circulatory stabilization and a subsequent decrease, analysis showed no difference for the crystalloid and resveratrol group (Fig. 3d; crystalloid: *P* = 0.28, *F*(1, 18) = 1.239; colloid: *P* = 0.034, *F*(4, 74) = 2.757; resveratrol: *P* = 0.308, *F*(1, 17) = 1.105)).

### Blood gas analysis

Blood gas analysis was performed on both intraflap venous blood and central venous blood at each time point beginning from T4. The analysis was structured into two distinct parts with an initial comparison of intraflap venous blood across different treatment groups, and a subsequent group-specific comparison between intraflap and central venous blood. Analysis of pre-shock and post-shock-phases were conducted separately. Pre-shock data were analyzed using one-way analysis of variance (ANOVA) to identify baseline differences in blood gas parameters. Starting from T5, with animals assigned to different treatment groups, data were analyzed using mixed-effects models employing the restricted maximum likelihood method. Post-hoc comparisons of individual time points were conducted using Šidák’s correction to adjust for multiple testing.

### Intraflap acid–base balance

**Fig. 4:**
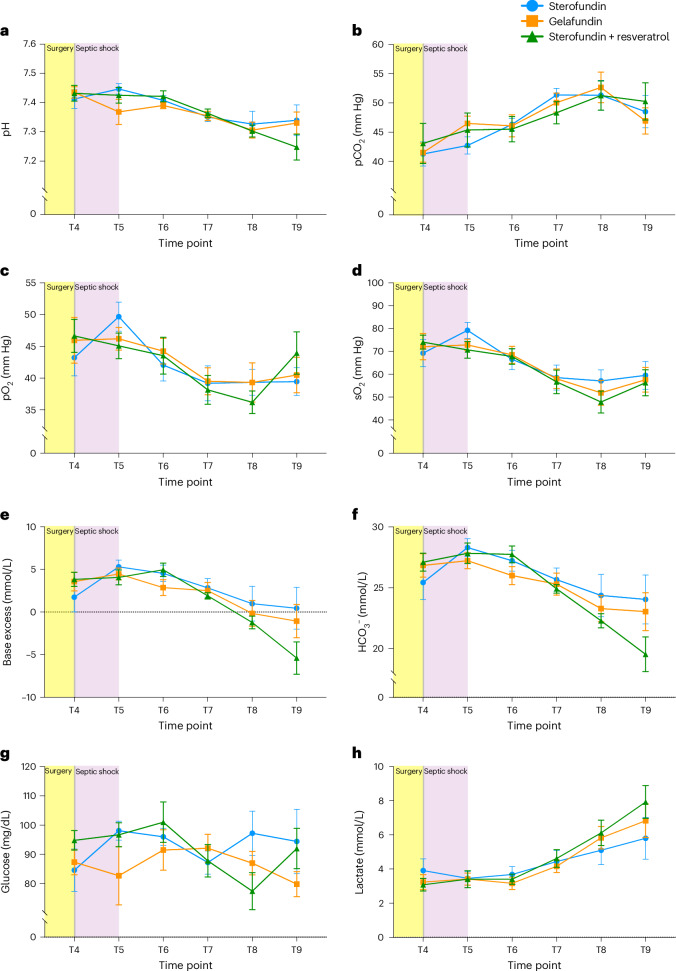
Intraflap acid–base balance. **a**–**h**, pH (**a**), pCO_2_ (**b**), pO_2_ (**c**), sO_2_ (**d**), base excess (**e**), HCO_3_⁻ (**f**), glucose (**g**) and lactate (**h**) from intraflap venous blood are plotted as line charts across multiple measurement time points from T4 to T9, starting immediately following reperfusion. The yellow area indicates the surgical procedure, whereas the violet area reflects the shock phase with subsequent assessment of 3 different treatment groups comparing a balanced crystalloid solution (Sterofundin, *n* = 10), a colloid (Gelafundin, *n* = 11) and a combination of crystalloid and resveratrol (Sterofundin + resveratrol, *n* = 10). Data represent mean ± standard error of the mean. Statistical analysis at T4 was performed using one-way ANOVA; subsequent analysis was performed using a mixed-effects model fit with restricted maximum likelihood. Statistical significance was set at *P < 0.05.

At T4, before the initiation of any therapeutic intervention, one-way ANOVA indicated no treatment effects, as shown in Fig. 4a–h (pH: *P* = 0.813, *F*(2, 26) = 0.209; pCO_2_: *P* = 0.86, *F*(2, 26) = 0.152; pO_2_: *P* = 0.693, *F*(2, 26) = 0.373; O_2_: *P* = 0.791, *F*(2, 26) = 0.237; base excess: *P* = 0.457, *F*(2, 26) = 0.808; HCO_3_^−^: *P* = 0.498, *F*(2, 26) = 0.716; glucose: *P* = 0.372, *F*(2, 26) = 1.028; lactate: *P* = 0.486, *F*(2, 24) = 0.745), confirming the absence of differences between groups at this stage. Subsequent analysis using a mixed effects model of various parameters including pH, pCO_2_, pO_2_, sO_2_, base excess, HCO_3_^−^ and lactate revealed a significant influence of time. Specifically, pH (*P* < 0.001, *F*(1.971, 51.24) = 12.11), pO_2_ (*P* < 0.001, *F*(3.068, 79.76) = 11.57), sO_2_ (*P* < 0.001, *F*(3.215, 83.58) = 19.98), HCO_3_^−^ (*P* < 0.001, *F*(1.511, 39.29) = 24.73) and base excess (*P* < 0.001, *F*(1.475, 38.35) = 22.19) exhibited a decrease, whereas pCO_2_ (*P* < 0.001, *F*(2.265, 58.89) = 6.551) and lactate (*P* < 0.001, *F*(1.466, 38.11) = 32.68) levels increased within the flap throughout the observation period. Glucose (*P* = 0.471, *F*(2.437, 63.37) = 1.449) levels fluctuated throughout the experiment without any clear trend toward either increase or decline. However, no significant effects were noted for treatment type or the interaction between time and treatment. Consequently, no differences among the therapy groups were observed regarding any acid–base balance parameters assessed.

### Intraflap electrolytes

**Fig. 5:**
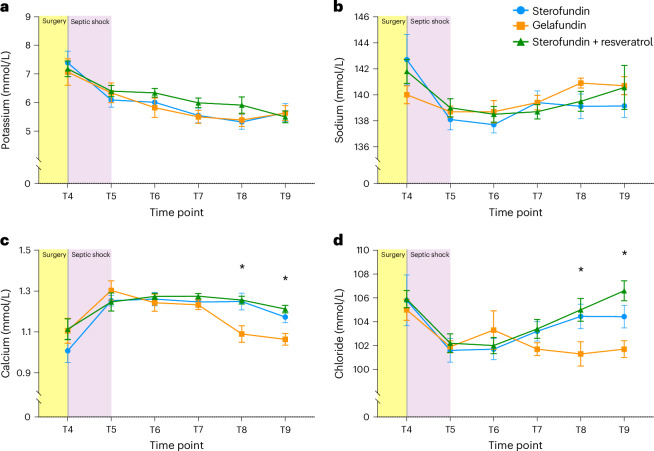
Intraflap electrolytes. **a**–**d**, Potassium (**a**), sodium (**b**), calcium (**c**) and chloride (**d**) from intraflap venous blood are plotted as line charts across multiple measurement time points from T4 to T9, starting immediately following reperfusion. The yellow area indicates the surgical procedure, whereas the violet area reflects the shock phase with subsequent assessment of 3 different treatment groups comparing a balanced crystalloid solution (Sterofundin, *n* = 10), a colloid (Gelafundin, *n* = 11) and a combination of crystalloid and resveratrol (Sterofundin + resveratrol, *n* = 10). Data represent mean ± standard error of the mean. Statistical analysis was performed using a mixed-effects model fit with restricted maximum likelihood, with post-hoc comparisons adjusted using Šidák’s correction. Statistical significance was set at *P < 0.05. Asterisks indicate individual time points with significant post-hoc differences.

As presented in Fig. 5a,b, mixed-effects modeling showed a significant overall effect of time for potassium (*P* = 0.002, *F*(1.963, 51.04) = 7.257) and sodium (*P* = 0.005, *F*(1.950, 50.21) = 6.009), indicating a decline during the shock phase, with sodium subsequently returning toward baseline levels during the therapy phase, without significant differences between treatment groups. Conversely, the mixed-effects analysis revealed variations in calcium (Fig. 5c, *P* = 0.001, *F*(8, 104) = 3.575) and chloride (Fig. 5d, *P* < 0.001, *F*(8, 104) = 4.212) levels, suggesting a significant influence of time and therapy. Time points showing significant differences in the post-hoc comparison, adjusted using Šidák’s correction, are marked in Fig. 5.

### Blood gas analysis—group-specific comparison of intraflap and central venous blood

Blood gas measurements specific to each treatment group were conducted from both the intraflap and central venous blood at each time point, enabling a comparison of localized, flap-specific changes against changes in systemic circulation in response to fluid resuscitation strategies.

### Acid–base balance—group-specific comparison of intraflap versus central venous blood

**Fig. 6:**
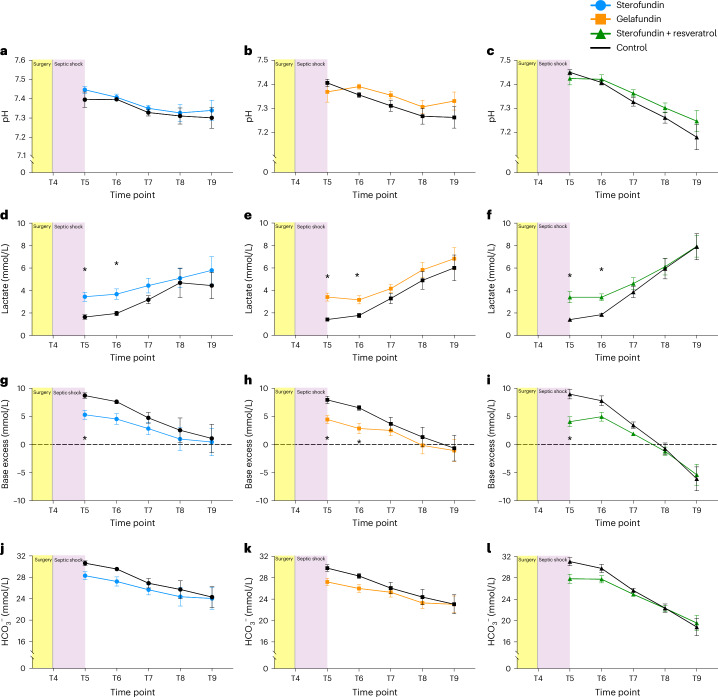
Acid–base balance—group-specific comparison of intraflap versus central venous blood. **a**–**l**, pH (**a**–**c**), lactate (**d**–**f**), base excess (**g**–**i**), and HCO_3_⁻ (**j**–**l**) are plotted as line charts. For each parameter, panels in the left, middle, and right columns correspond to the Sterofundin, Gelafundin, and Sterofundin + resveratrol groups, respectively. Intraflap and central venous blood were sampled simultaneously across multiple measurement time points from T5 to T9, starting immediately following circulatory stabilization. The yellow area indicates the surgical procedure, whereas the violet area reflects the shock phase. Assessment of flap-specific changes compared with the corresponding control (central venous blood) was performed for all 3 treatment groups including a balanced crystalloid solution (Sterofundin, *n* = 10), a colloid (Gelafundin, *n* = 11) and a combination of crystalloid and resveratrol (Sterofundin + resveratrol, *n* = 10). Data represent mean ± standard error of the mean. Statistical analysis was performed using a mixed-effects model fit with restricted maximum likelihood, with post-hoc comparisons adjusted using Šidák’s correction. Statistical significance was set at *P < 0.05. Asterisks indicate individual time points with significant post-hoc differences.

As presented in Fig. 6a–c, mixed-effects analysis with post-hoc comparisons with Šidák’s correction showed no significant differences in pH between intraflap venous blood and central venous blood across all three experimental groups. However, lactate levels (Fig. 6d–f) were significantly elevated within the free flap following circulatory stabilization compared with central venous blood (Fig. 6d, T5: *P* = 0.007, *t* = 4.013, d.f. 13.19; T6: *P* = 0.025, *t* = 3.404, d.f. 12.16; Fig. 6e, T5: *P* = 0.001, *t* = 5.32, d.f. 11.66; T6: *P* = 0.021, *t* = 3.418, d.f. 13.74; Fig. 6f, T5: *P* = 0.014, *t* = 3.9, d.f. 9.99; T6: *P* = 0.002, *t* = 4.79, d.f. 12.2). This elevation persisted for approximately 1 h before lactate levels in the flap gradually aligned with those in the central venous blood. Conversely, a reversed pattern was noted for base excess as shown in Fig. 6g–h, with significantly lower levels within the free flap immediately after circulatory stabilization (Fig. 6g, T5: *P* = 0.011, *t* = 3.69, d.f. 14.87; Fig. 6h, T5: *P* = 0.008, *t* = 3.69, d.f. 17.86; T6: *P* = 0.016, *t* = 3.62, d.f. 12.76; Fig. 6i, T5: *P* = 0.003, *t* = 4.09, d.f. 17.97) and equal levels at T9 compared with central venous blood. In addition, no differences in bicarbonate levels (Fig. 6j,k) were found between intraflap venous and central venous blood across any of the experimental groups.

### Electrolytes—group-specific comparison of intraflap versus central venous blood

**Fig. 7:**
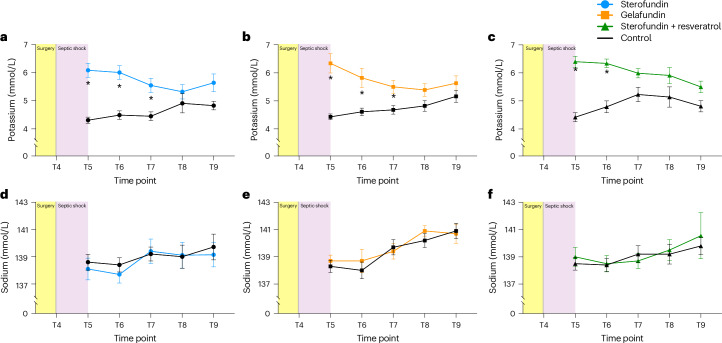
Electrolytes—group-specific comparison of intraflap versus central venous blood. **a**–**f**, Potassium (**a**–**c**) and sodium (**d**–**f**) are plotted as line charts. For each parameter, panels in the left, middle, and right columns correspond to the Sterofundin, Gelafundin, and Sterofundin + resveratrol groups, respectively. Intraflap and central venous blood were sampled simultaneously across multiple measurement time points from T5 to T9, starting immediately following circulatory stabilization. The yellow area indicates the surgical procedure, whereas the violet area reflects the shock phase. Assessment of flap-specific changes compared with the corresponding control (central venous blood) was performed for all 3 treatment groups including a balanced crystalloid solution (Sterofundin, *n* = 10), a colloid (Gelafundin, *n* = 11) and a combination of crystalloid and resveratrol (Sterofundin + resveratrol, *n* = 10). Data represent mean ± standard error of the mean. Statistical analysis was performed using a mixed-effects model fit with restricted maximum likelihood, with post-hoc comparisons adjusted using Šidák’s correction. Statistical significance was set at *P < 0.05. Asterisks indicate individual time points with significant post-hoc differences in **a**–**c**.

Mixed-effects analysis confirmed a significant impact of therapy (Fig. 7a, *P* < 0.001, *F*(1, 18) = 24.13; Fig. 7b, *P* < 0.001, *F*(1, 18) = 18.97; Fig. 7c, *P* < 0.001, *F*(1, 18) = 24.20), as well as the interaction between time and therapy (Fig. 7a, *P* = 0.003, *F*(4, 64) = 4.487; Fig. 7b, *P* = 0.001, *F*(4, 72) = 4.985; Fig. 7c, *P* < 0.001, *F*(4, 72) = 5.441) on potassium levels across all 3 experimental groups. Asterisks in Fig. 7a–c indicate individual time points with significant post-hoc differences between intraflap venous and central venous potassium levels after Šidák’s correction. Immediately following circulatory stabilization (T6), intraflap venous potassium levels were significantly elevated in all three groups compared with those in central venous blood. During the subsequent observation period (T7–T9), potassium levels within the flap normalized compared with central venous levels. As presented in Fig. 7d–f, there was no difference in sodium levels between intraflap venous and central venous blood in any of the three experimental groups. Throughout the experiment, sodium levels increased in both intraflap and central venous blood across all groups.

### Blood gases—group-specific comparison of intraflap versus central venous blood

**Fig. 8:**
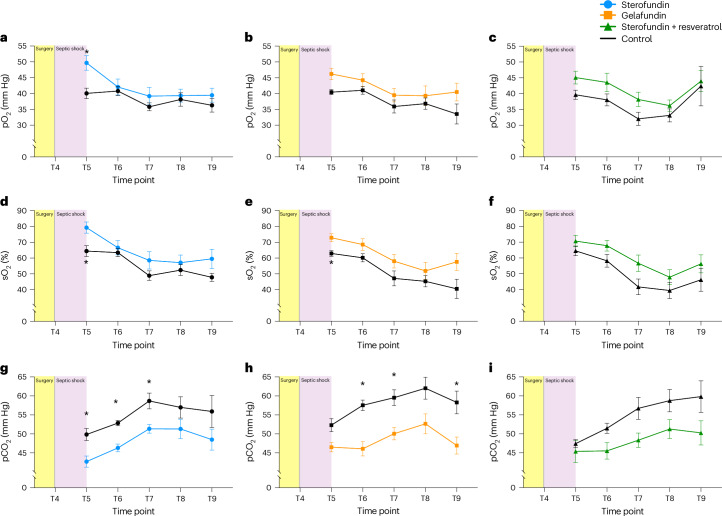
Blood gases—group-specific comparison of intraflap versus central venous blood. **a**–**i**, pO_2_ (**a**–**c**), sO_2_ (**d**–**f**) and pCO_2_ (**g**–**i**) are plotted as line charts. For each parameter, panels in the left, middle, and right columns correspond to the Sterofundin, Gelafundin, and Sterofundin + resveratrol groups, respectively. Intraflap and central venous blood were sampled simultaneously across multiple measurement time points from T5 to T9, starting immediately following circulatory stabilization. The yellow area indicates the surgical procedure, whereas the violet area reflects the shock phase. Assessment of flap-specific changes compared with the corresponding control (central venous blood) was performed for all 3 treatment groups including a balanced crystalloid solution (Sterofundin, *n* = 10), a colloid (Gelafundin, *n* = 11) and a combination of crystalloid and resveratrol (Sterofundin + resveratrol, *n* = 10). Data represent mean ± standard error of the mean. Statistical analysis was performed using a mixed-effects model fit with restricted maximum likelihood, with post-hoc comparisons adjusted using Šidák’s correction. Statistical significance was set at *P < 0.05. Asterisks indicate individual time points with significant post-hoc differences.

Analysis indicated significant differences in pO_2_ between intraflap and central venous blood, with the crystalloid group showing increases due to both time and therapy effects (Fig. 8a, *P* = 0.049, *F*(4, 64) = 2.520), and the colloid group showing increases attributed only to therapy (Fig. 8b, *P* = 0.032, *F*(1, 18) = 5.427). Initially increased pO2 levels at T5 subsequently returned to baseline levels. By contrast, no differences were observed in the resveratrol group compared with control (Fig. 8c). As presented in Fig. 8d–f, these results align with intraflap blood consistently exhibiting higher sO_2_ levels compared with control (crystalloid: *P* = 0.072, *F*(1, 18) = 3.664; colloid: *P* = 0.01, *F*(1, 18) = 8.133; resveratrol: *P* = 0.043, *F*(1, 18) = 4.759). Therapy further significantly influenced pCO_2_ levels in all 3 groups, with intraflap venous blood consistently showing lower pCO_2_ levels than central venous blood (Fig. 8g, *P* = 0.001, *F*(1, 18) = 15.53; Fig. 8h, *P* < 0.001, *F*(1, 18) = 24.17; Fig. 8i, *P* = 0.018, *F*(1, 18) = 6.772).

### Therapy group-specific correlations of metabolism and perfusion

Lastly, the correlations between lactate and StO_2_, base excess and NPI, and THI and TWI were specifically analyzed. Lactate and StO_2_ represent the balance between oxygen delivery and consumption, whereas the correlation between base excess and NPI was investigated to assess the metabolic compensation in response to altered perfusion. The correlation of THI and TWI, as indicators of hemoglobin levels and tissue hydration, reflect fluid dynamics within the flap.

### Correlation of StO_2_ and lactate levels

**Fig. 9:**
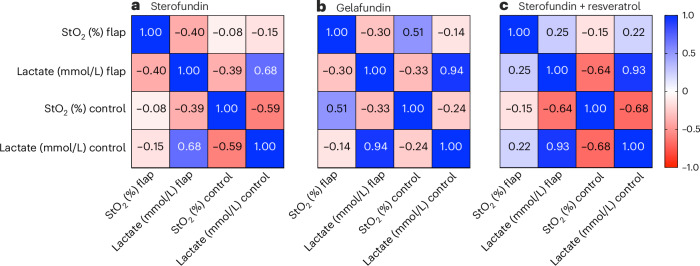
Correlation of StO_2_ and lactate levels. **a**–**c**, Correlation matrices showing the relationship between StO_2_ and lactate of the free flap, as well as StO_2_ and lactate of the control under different treatment conditions: crystalloid (Sterofundin; StO_2_ *n* = 47; lactate *n* = 46) (**a**), colloid (Gelafundin; StO_2_ *n* = 52; lactate *n* = 49) (**b**), and crystalloid and resveratrol (Sterofundin + resveratrol; StO_2_ *n* = 50/43; lactate *n* = 50/49) (**c**). The color gradient indicates the strength and direction of the correlation, with blue indicating positive correlations and red indicating negative correlations. The values within each cell represent the Pearson correlation coefficient. Group-specific comparison of the three different treatment groups involved assessment of all measurements following circulatory stabilization (T5–T9).

As illustrated in Fig. 9a, the crystalloid group displayed a moderate negative correlation between StO_2_ and lactate levels within the free flap (*r* = −0.399, *P* = 0.006), compared with a strong negative correlation observed in the control region (*r* = −0.592, *P* < 0.001). Conversely, in the resveratrol group, the central venous control demonstrated a strong negative correlation (*r* = −0.679, *P* < 0.001), whereas only a weak, nonsignificant positive correlation was found within the free flap (*r* = 0.251, *P* = 0.079) (Fig. 9c). The colloid group showed a weak negative correlation in the flap (*r* = −0.296, *P* = 0.039), while the control region showed only a weak, nonsignificant negative correlation (*r* = −0.243, *P* = 0.092) (Fig. 9b).

### Correlation of NPI and base excess

**Fig. 10:**
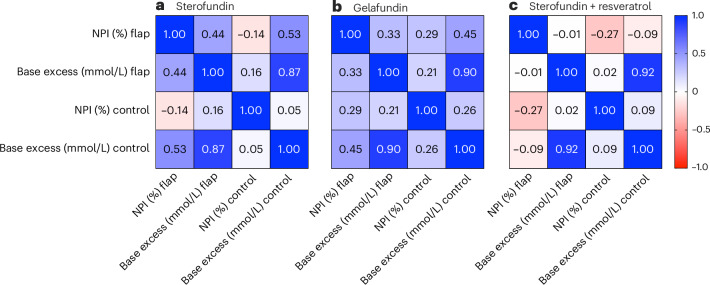
Correlation of NPI and base excess. **a**–**c**, Correlation matrices showing the relationship between NPI and base excess of the free flap, as well as NPI and base excess of the control under different treatment conditions: crystalloid (Sterofundin; NPI *n* = 47; base excess *n* = 46) (**a**), colloid (Gelafundin; NPI *n* = 52; base excess *n* = 49) (**b**), and crystalloid and resveratrol (Sterofundin + resveratrol; NPI *n* = 50/43; base excess *n* = 50) (**c**). The color gradient indicates the strength and direction of the correlation, with blue indicating positive correlations and red indicating negative correlations. The values within each cell represent the Pearson correlation coefficient. Group-specific comparison of the three different treatment groups involved assessment of all measurements following circulatory stabilization (T5–T9).

In the crystalloid group, a positive correlation was noted between NPI and base excess in the free flap (Fig. 10a, *r* = 0.44, *P* = 0.002), contrasting with a very weak positive correlation in the control (Fig. 10a, *r* = 0.04, *P* = 0.757). Meanwhile, the colloid group exhibited a moderate positive correlation in the free flap (Fig. 10b, *r* = 0.33, *P* = 0.023), and a less pronounced but still positive correlation in controls (Fig. 10b, *r* = 0.26, *P* = 0.071). By contrast, the resveratrol group showed no correlation between NPI and base excess in both free flap (Fig. 10c, *r* = −0.01, *P* = 0.965) and control tissues (Fig. 10c, *r* = 0.09, *P* = 0.549).

### Correlation of THI and TWI

**Fig. 11:**
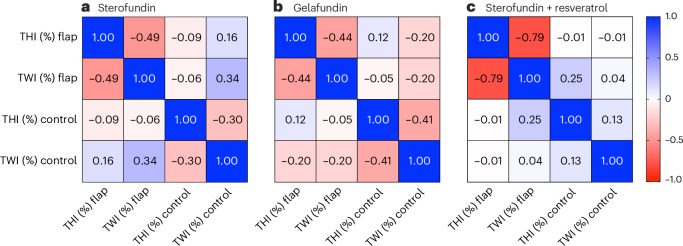
Correlation of THI and TWI. **a**–**c**, Correlation matrices showing the relationship between THI and TWI of the free flap, as well as THI and TWI of the control under different treatment conditions: crystalloid (Sterofundin; THI *n* = 47; TWI *n* = 47) (**a**), colloid (Gelafundin; THI *n* = 52; TWI *n* = 52) (**b**), and crystalloid and resveratrol (Sterofundin + resveratrol; THI *n* = 50/43; TWI *n* = 50/43) (**c**). The color gradient indicates the strength and direction of the correlation, with blue indicating positive correlations and red indicating negative correlations. The values within each cell represent the Pearson correlation coefficient. Group-specific comparison of the three different treatment groups involved assessment of all measurements following circulatory stabilization (T5–T9).

Across all treatment groups, statistically significant negative correlations between THI and TWI were noted in the free flap tissues as presented in Fig. 11a–c. The strongest negative correlation was observed in the resveratrol group (*r* = −0.786, *P* < 0.001), followed by moderate negative correlations in the crystalloid (*r* = −0.487, *P* = 0.001) and colloid groups (*r* = −0.445, *P* = 0.001). By contrast, the control tissues exhibited moderate negative correlations in the crystalloid (*r* = −0.304, *P* = 0.038) and colloid (*r* = −0.410, *P* = 0.003) groups. Notably, the control tissues of the resveratrol group showed no significant correlation (*r* = 0.128, *P* = 0.414).

## Discussion

The primary challenge in microvascular free flap transfer remains ensuring flap survival, particularly in the context of reconstructive head and neck surgery. Beyond complications associated with impaired perfusion^3^, postoperative infection leading to sepsis has been identified as risk factor for late flap failure^7,8^. Thus, effective monitoring and timely intervention strategies are essential to ensure flap survival and enhance patient outcomes. This study aimed to investigate the impact of septic shock on free flap vitality using a large animal model that reflects clinical conditions. Microvascular free tissue transfer was performed in pigs owing to their physiological similarity to human anatomy and immune response^12,13^. Septic shock was induced by LPS infusion, resulting in a severe systemic reaction potentially compromising the free flap. Subsequently, the efficacy of three different fluid resuscitation strategies, including a crystalloid solution, a colloid and a combination of crystalloid with resveratrol were evaluated. Free flap vitality was continuously monitored using HSI and further complemented by blood gas analyses comparing intraflap venous blood with simultaneously collected central venous blood samples. Physiological changes within the free flap were evaluated in relation to systemic alterations to determine the direct effects of various volume resuscitation strategies on free flap viability during septic shock. Microvascular free flap transfer, representing the gold standard in reconstructive surgery, remains an extensive and complex procedure. Despite the advancements leading to high survival rates, the complexity of the procedure increases with flap difficulty and patient comorbidities^1,3^. Harvesting of the flap results in unavoidable local tissue damage. Ischemia following the dissection of the flap’s pedicle vessels persists until oxygenation is restored upon successful re-anastomosis. HSI can provide detailed visualization of each procedural step. During the surgical intervention, a notable decrease in StO_2_ was observed, attributable to vascular dissection and relocation of the flap to the axillary region, directly affecting flap perfusion. However, StO_2_ increased thereafter following successful reanastomosis and reperfusion, indicating effective restoration of arterial inflow and perfusion. Similarly, THI initially decreased due to the reduced blood volume but increased after reperfusion. This overcompensation in THI is a result of strong arterial inflow coupled with initially delayed venous outflow gradually adapting to new physiological flap conditions. The duration of this intraoperative ischemia, typically ranging from 15 to 30 min, depends largely on surgical expertise. Although muscle tissue is highly tolerant to ischemia^14,15^, our previous research has shown induction of anaerobic glycolysis due to oxygen depletion resulting in elevated lactate and potassium levels within the flap after short ischemic periods^12^. The TWI further increased throughout surgery, probably due to an impairment in venous outflow. The subsequent normalization of water levels by T4, following the re-establishment of venous outflow, highlights the critical role of efficient venous drainage in diminishing tissue edema and avoiding flap congestion. Taken together, these intraoperative observations describe the characteristic pathophysiological changes of free flaps during ischemia and the early reperfusion phase.

As the surgical field expands, the risk of postoperative infection elevates with increasing wound size. Nevertheless, the research on intraoperative and postoperative fluid management has predominantly focused on patients undergoing intra-abdominal surgeries^16,17^, with limited studies dedicated to head and neck procedures. The uniqueness of this study is thereby emphasized, which uses an innovative approach of venous flap pedicle catheterization. This experimental model has been previously validated for assessing metabolic changes within the free flap during the hemorrhagic shock and was now adapted for the investigation of septic shock^13^.

During the shock phase and the subsequent monitoring, absolute HSI values showed no differences among the three treatment groups. However, by normalizing these values against control measurements from abdominal skin at each time point, nuanced effects of septic shock and fluid resuscitation on both the free flap and the organism as a whole were detected. This normalization minimized interanimal variability and enabled comparison of local tissue responses. Notably, significant differences in StO_2_ were observed among the therapeutic groups, with the crystalloid group demonstrating the highest levels within the free flap, suggesting balanced crystalloid solutions to optimize oxygen delivery by enhancing microcirculatory perfusion—a finding consistent with our previous research on hemorrhagic shock^13^. This finding is further supported by a negative correlation between StO_2_ and lactate within the free flap and control. Consequently, compared with the colloid solution and resveratrol, the crystalloid solution seems to most effectively restore sufficient oxygenation within the flap, thereby reducing the impact of the septic shock. However, increased TWI in the crystalloid group indicated a potential fluid overload, which could lead to flap congestion or edema, therefore highlighting the critical need for precise intraoperative fluid management, especially under septic conditions. Overhydration during head and neck cancer surgeries poses a risk for increased complication rates, while inadequate resuscitation could lead to postoperative flap thrombosis^18,19^. Current evidence favors crystalloids over colloids for fluid resuscitation, with recommendations limiting crystalloid volumes to 130 mL/kg over 24 h (ref. ^20^). The finding of higher StO_2_ levels in the crystalloid group supports the use of balanced crystalloid solutions to sustain microcirculatory perfusion during septic shock; however, there remains a risk of overhydration. Nevertheless, lower TWI values in the resveratrol group indicate a more efficient fluid management without fluid overload, potentially reducing postoperative edema and enhancing flap survival. Although all groups demonstrated negative correlations between TWI and THI, suggesting a balance between hydration and blood volume, this potential in enhancing fluid dynamics within tissue at risk was most pronounced in the resveratrol group, as reflected by the strongest negative correlation. Notably, this correlation was absent in control tissues, implying that resveratrol’s effect was flap-specific rather than systemic. Although the present study was not designed to elucidate mechanistic pathways, resveratrol is known to exert anti-inflammatory and antioxidative effects, including inhibition of NF-κB signaling and reduction of reactive oxygen species, which can stabilize endothelial barrier function and thereby limit capillary leakage. Resveratrol has further been reported to support endothelial integrity and microvascular perfusion through endothelial nitric oxide synthase activation, potentially reducing interstitial fluid accumulation^21,22^. These mechanisms remain hypothetical in the context of free flap physiology, but they may contribute to the lower TWI values and the more pronounced negative THI–TWI correlation seen in the resveratrol group. In addition, the absence of lymphatic drainage in free flaps increases the risk of interstitial edema, as blood flow within the flap typically reduces to half its normal rate after surgery and requires an extended period to normalize.

Although resveratrol demonstrated a weaker impact on StO_2_ compared with crystalloid solutions, it maintained a consistent NPI, suggesting stable flap perfusion and, therefore, a reduced risk of thrombosis and vessel occlusion. This is particularly relevant given that hemodynamic fluctuations contribute to thrombogenesis as part of Virchow’s triad^23^. However, despite its antioxidant and anti-inflammatory properties, resveratrol alone may not be able to adequately restore oxygenation in compromised tissues during septic shock. Conversely, the colloid group showed the lowest StO_2_ levels, suggesting reduced oxygen delivery and perfusion after colloid-based fluid administration. Although an initial increase in the NPI was observed, it soon decreased, indicating that the improvement in perfusion was only temporary.

Moreover, the comparison of free flap tissue with abdominal skin revealed consistently lower StO_2_ levels in the flap reflecting the transferred tissue’s vulnerability to ischemic stress, particularly in systemic inflammatory conditions. The inflammatory response from surgical tissue damage and systemic sepsis results in an increased risk of malperfusion within the flap. The lack of oxygen supply leads to the transition to anaerobic metabolism with reduced ATP production, lactate accumulation and, hence, intracellular acidosis. Acidosis further destabilizes lysosomal membranes with subsequent enzyme leakage, cytoskeleton breakdown, and Na^+^/K^+^ ATPase activity inhibition^24^. Finally, cellular swelling occurs due to intracellular sodium and subsequent water accumulation. The combined effects of oxidative stress and cell swelling exacerbate malperfusion, as both factors collectively intensify the impairment of tissue viability. The study further found no significant differences in TWI or THI between the groups, indicating that the employed fluid resuscitation strategies did not alter the blood volume or hydration status within the flap. Stable THI suggests effective blood outflow management, preventing tissue swelling. Similarly, the lack of significant changes in TWI or THI between the flap and control tissue points to a consistent therapeutical impact of systemic changes on both the flap and the central body, negating any flap-specific effects from the employed fluid resuscitation therapies.

Despite no significant differences in pH, pCO_2_, pO_2_, sO_2_, base excess, bicarbonate, glucose and lactate levels after shock between the groups, different patterns regarding the local metabolic profiles within the flaps were observed. Notably, lactate levels in the free flap were significantly increased compared with central venous blood across all treatment groups following circulatory stabilization. This increase persisted for 1 h before gradually aligning with central venous levels, reflecting localized metabolic stress and delayed perfusion recovery within flap tissue. These results are consistent with well-understood anoxic conditions triggered by the interruption of blood flow, leading to anaerobic metabolism, accumulation of lactate and a decrease in intracellular pH. In addition, oxygen depletion reduces adenosine triphosphate levels, increases calcium ion concentration and triggers the release of pro-inflammatory mediators. The extent of damage during primary ischemia correlates directly with the duration of the ischemic period^25^. Upon reperfusion, tissues damage is further triggered by reperfusion injury, involving activation of programmed cell death, endothelial dysfunction, transcriptional changes and activation of immune responses^26^. Conversely, base excess levels were lower in the flap immediately after circulatory stabilization, aligning with the findings of metabolic acidosis due to the accumulation of acidic metabolic byproducts during the ischemic phase. Similar base excess levels within the flap and central venous blood by T9 suggest a gradual normalization of metabolic conditions upon restoration of perfusion. Considering the positive correlation between NPI and base excess in free flap tissues in the crystalloid group, previous findings of improved perfusion and metabolic balance upon administration of crystalloid fluids are supported. By contrast, the resveratrol group showed no correlation between NPI and base excess in both free flap and control tissues.

A methodological limitation of the experimental setup is the absence of blinding during data collection and analysis. Although treatment-group allocation was randomized before shock induction, blinding was not feasible once the resuscitation fluids were administered, as they were visually distinguishable and required specific handling. Nevertheless, given that perfusion and metabolic parameters were obtained using automated and objective measurement systems, any potential observer bias should be minimal.

Intracellular acidosis diminishes Ca^2+^ excretion, leading to an intracellular Ca^2+^ accumulation and, hence, activating Ca^2+^-dependent proteases such as calpains^24,27^. This process is reflected in this study’s venous blood gas analysis, showing significantly elevated intraflap venous potassium levels compared with central venous potassium levels in all experimental groups followed by a subsequent normalization. Therefore, the concept of cellular distress during the initial shock phase resulting in a release of intracellular contents is supported. In addition, calcium and chloride levels were significantly increased in crystalloid and resveratrol animals compared with colloid, while sodium levels remained stable, showing no difference either between groups nor between flap and central venous blood. During reperfusion, as oxygen and pH levels normalize, previously ischemic cells are paradoxically harmed due to intracellular calcium, which activates calpains^26^. The restoration of normoxemia further triggers the production and release of reactive oxygen species and thereby a reduction in antioxidant capacity. However, convergence of metabolic parameters with central nervous levels reflects the restoration of cellular integrity and flap viability over time, as perfusion is re-established. Flap-specific effects are indicated by a significant decrease in calcium levels over time in the colloid group, while significant increases in chloride levels in the crystalloid and resveratrol groups can be attributed to the constitution of the fluids used for resuscitation, with Sterofundin containing approximately 10–12 mmol/L more chloride ions than Gelafundin. Although these chloride shifts were within a physiologically acceptable range, their potential clinical impact should be considered. High-chloride fluids have been associated with hyperchloremic acidosis, reduced renal perfusion and an increased risk of acute kidney injury in septic and critically ill patients^28^. In the context of microvascular reconstruction, excessive chloride loads may exacerbate interstitial fluid accumulation and endothelial dysfunction, thereby influencing flap edema and microcirculatory performance. While our model does not allow long-term assessment of these consequences, the observed group differences underscore the importance of balanced electrolyte composition in resuscitation fluids when tissue perfusion is critically compromised. These findings further illustrate that while systemic responses to septic shock and resuscitation are generally consistent across treatment groups, localized responses within the flap can vary significantly, influenced by the specific dynamics of fluid therapy and ischemic conditions.

Furthermore, significant differences in pO_2_ between intraflap venous and central venous blood were noted, particularly in the crystalloid and colloid groups, showing initially increased pO_2_ levels at T5 that subsequently returned to baseline. This observation aligns with consistently higher sO_2_ levels in intraflap compared with central venous blood across all groups, suggesting effective oxygenation at the flap site. Conversely, systemic circulatory centralization impairs tissue perfusion in peripheral muscle and skin, thereby enhancing anaerobic metabolism more pronouncedly than in the flap owing to the absence of neural innervation making flap tissue less susceptible to sympathetically induced vasoconstriction. Moreover, the consistently lower pCO_2_ levels in intraflap venous blood suggest enhanced tissue perfusion, aligning with findings from other studies^29–32^. The cellular response to oxygen depletion, characterized by anaerobic metabolism and reactive oxygen species generation, is well documented. The significant variations in pO_2_ and consistent sO_2_ levels underscore the efficacy of the employed fluid resuscitation strategies in maintaining adequate oxygenation within the flap. This alignment of our findings with established physiological responses validates the robustness of our experimental approach and validates it for further experiments.

Despite the strengths of this experimental model, certain limitations need to be considered. The use of LPS-induced shock in young, otherwise healthy animals provides a controlled and reproducible experimental setting, but it cannot fully reflect the complexity of clinical sepsis, which typically evolves over hours to days and is influenced by pre-existing comorbidities, variable inflammatory responses and prolonged hemodynamic instability. Likewise, the short post-resuscitation observation period provides insights solely into the immediate post-resuscitation period of early metabolic and microcirculatory recovery and does not capture potential long-term complications. In addition, the necessity of a catheterized venous outflow with continuous retransfusion creates a physiological setting that differs from routine free flap surgery, even though it enables high-resolution, flap-specific measurements unattainable in the clinical situation. Nevertheless, within the constraints inherent to experimental research on microvascular reconstruction under septic conditions, the presented model offers a robust platform for assessing the impact of different resuscitation strategies.

## Conclusion

The present study provides comprehensive insights into the effects of septic shock on microvascular free flap vitality by using an advanced large animal model. Our findings indicate that fluid resuscitation strategies, particularly crystalloid solutions, are critical in optimizing flap perfusion and managing systemic responses to septic shock. The research highlights the vulnerable balance required in fluid management to enhance flap survival without inducing fluid overload, emphasizing the importance of individually adapted treatment approaches. In addition, the study confirms the complex interplay between local flap conditions and systemic changes, underscoring the necessity of precise monitoring and timely interventions to mitigate complications such as ischemia and edema in flap surgeries. Future research should continue to refine these strategies to improve outcomes in reconstructive surgery, integrating both systemic and localized treatment considerations.

## Methods

### Experimental design and ethical statement

This prospective, randomized trial was conducted with 31 juvenile male pigs (*Sus scrofa domestica*) weighing 31 ± 2.1 kg and aged 10–12 weeks). Three additional animals were excluded before initiation of the experimental protocol because of cardiovascular instability. The study received approval of the State and Institutional Animal Care Committee Rhineland Palatine (approval number G21-1-080; date of approval, 22 November 2021) and was performed at the Department of Anesthesiology, University Medical Centre Mainz. All experimental procedures adhered strictly to the German Animal Protection Law, the ARRIVE guidelines, and Directive 2010/63/EU of the European Parliament on the protection of animals used for scientific purposes, thereby upholding high ethical and scientific standards.

### Anesthesia and surgical procedure

The experimental protocol regarding animal handling, instrumentation, anesthesia and surgical procedure has been described in great detail in previous publications by our group^12,33^. In brief, the animals were fasted for 6 h to prevent aspiration during intubation, while unrestricted access to water was maintained. Sedation was administered via intramuscular injections of azaperone (4 mg/kg) and ketamine (4 mg/kg). Following sedation, the animals were transported to the laboratory under continuous monitoring by a veterinarian. Anesthesia was initiated by intravenous injection of fentanyl (4 µg/kg), propofol (4 mg/kg) and atracurium (0.5 mg/kg) and maintained throughout the procedure with a continuous infusion of propofol (8–12 mg/kg/h) and fentanyl (0.1–0.2 µg/kg/h). The animals were ventilated with a volume-controlled Evita 2 ventilator (Draeger). Vascular access for advanced hemodynamic monitoring was established via catheterization of the right femoral artery using a pulse contour cardiac output system (PiCCO, Pulsion Medical Systems). Additional central catheters were placed in the left femoral vein and artery for thermodilution measurements and fluid management, with ultrasound guidance ensuring precise placement. Finally, the surgical protocols involved preparation of a myocutaneous gracilis flap from the right thigh^34–36^, followed by the free tissue transfer to the right axilla. The recipient site was prepared simultaneously to minimize anesthesia duration. Microvascular anastomosis was performed according to our established technique with an end-to-side arterial anastomosis between the flap pedicle artery and the axillary artery^12,13^. The surgical setup and procedural steps are illustrated in Supplementary Fig. 1. For flap-specific metabolic monitoring, the venous pedicle was catheterized using a venous catheter system (LOGICATH, ICU Medical). The catheter allowed continuous venous outflow from the flap, which drained into a standard blood transfusion bag containing citrate. Throughout the whole experiment the collected blood volume was quantified, filtrated to avoid embolism and retransfused (CompoFlow RCC, Fresenius Kabi). At each measurement time point, additional flap venous samples were obtained by briefly interrupting the continuous drainage. A total of 1 ml of blood was withdrawn for blood gas analysis and 3 ml for centrifugation and serum storage. Catheter patency was maintained by a single intravenous bolus of 5,000 IU heparin administered immediately after restoration of arterial inflow, preventing catheter occlusion without influencing systemic coagulation over the study period.

### Septic shock, fluid resuscitation and measurement time points

Following the surgical phase (T1 to T4), septic shock was induced using a continuous infusion of LPS (*Escherichia coli* serotype O111:B4, Sigma-Aldrich). The induction protocol began with a high dose of 150 μg/kg/h for 30 min (T4 to T5), transitioning to a maintenance dose of 15 μg/kg/h throughout the experiment. Starting at T5, fluid resuscitation therapy was administered using three different strategies: (1) a balanced crystalloid solution (Sterofundin ISO, B. Braun SE), (2) a colloidal solution (Gelafundin ISO 4%, B. Braun SE) and (3) a combination of Sterofundin ISO with resveratrol. Group allocation was performed by simple randomization using a lottery-draw system at the beginning of each experiment. Investigators were blinded to the assigned treatment group until the end of the surgical and shock induction phase. After initiation of the respective resuscitation regimen, blinding was no longer feasible, as the administered fluids were visually distinguishable and required specific handling. Circulatory stabilization, defined as a mean arterial pressure >60 mm Hg, marked time point T6. Monitoring continued throughout a 3-h observation period, with assessments recorded at T7 (1 h after stabilization), T8 (2 h after stabilization) and T9 (3 h after stabilization). Upon the completion of the observation period, the animals were euthanized using an injection of propofol followed by potassium chloride. Measurement time points are comprehensively depicted in Supplementary Table 1

### HSI

HSI measurements were conducted using the TIVITA Tissue System (Diaspective Vision), a device extensively detailed in our prior research^37,38^. This system generates a comprehensive data cube with two spatial dimensions (resolution: 0.1 mm per pixel at a 50-cm distance) and a spectral dimension (resolution: 5 nm), capturing data beyond the visible spectrum up to 740 nm. This advanced technology processes molecular signatures, particularly focusing on hemoglobin, oxygenated hemoglobin and water content, to provide in-depth physicochemical insights. The operational procedure includes a 10-s recording phase followed by an 8-s processing period to generate a true-color RGB image and four pseudocolor images. These images quantitatively represent various physiological parameters such as StO_2_ (0–100%), NPI (0–1), THI (0–1) and TWI (0–1) enabling the evaluation of microcirculatory dynamics and tissue health. Specifically, StO_2_ primarily measures venous oxygen saturation, typically ranging from 50% to 70% in healthy tissue. The NPI measures blood flow quality in deeper tissues (4–6 mm), while THI and TWI quantify the concentrations of hemoglobin and water. The experimental setup is depicted in Supplementary Fig. 2.

### Blood gas analysis

Blood samples were collected simultaneously from both intraflap venous and central venous catheters immediately after flap reperfusion and at regular intervals from T4 to T9. These samples were analyzed using the ABL 90 Flex blood gas analyzer (Radiometer) to assess pH, metabolites, blood gases and electrolytes.

### Statistical analysis

Statistical analyses were performed using GraphPad Prism 9.0 (GraphPad Software), Excel 16.76 (Microsoft) and SPSS Statistics 29 (IBM Deutschland). ChatGPT 4.0 assisted with proofreading. All numerical data are presented as mean ± standard error of the mean, with statistical significance established at *P* < 0.05. During the surgical phase, no significant differences in HSI free flap values were detected among treatment groups, as determined by one-way ANOVA with repeated measures. Consequently, all animals were considered a single group for initial analyses. The effect of surgery was assessed by comparing each intrasurgery time point (T2–T4) against the baseline (T1) for the surgery group using multiple *t*-tests.

Post-shock effects on time, therapy, and their interaction on the free flap were analyzed using a mixed-effects model with restricted maximum likelihood. This approach was selected over two-way ANOVA to accommodate missing values at certain time points, thus preventing the exclusion of entire datasets. Post-hoc comparisons used Šidák’s correction to adjust for multiple testing and to identify specific differences between groups or time points. HSI data were subsequently normalized against control values (abdominal skin) with the control mean set at 100% to evaluate the flap-specific impact of therapy. Finally, analysis included a group-specific evaluation of flap versus control for each therapy group. Blood gas analysis at T4 involved a one-way ANOVA to assess baseline differences in blood gas parameters among therapy groups. To investigate differences between central venous and intraflap venous blood gas parameters across therapy groups and over time, the same mixed-effects model a described above was used. Correlations between variables were determined using Pearson correlation coefficient separately for each treatment group using all available measurements obtained after circulatory stabilization (T5–T9). Depending on the respective analysis, paired flap and control parameters were included, and only observations with complete data for both variables were entered. The corresponding sample sizes are reported in the figure legends.

### Reporting summary

Further information on research design is available in the Nature Portfolio Reporting Summary linked to this article.

## Online content

Any methods, additional references, Nature Portfolio reporting summaries, source data, extended data, supplementary information, acknowledgements, peer review information; details of author contributions and competing interests; and statements of data and code availability are available at 10.1038/s41684-026-01747-0.

## Supplementary information

Supplementary Information Supplementary Figs. 1 and 2 and Table 1.

Reporting Summary

## Data Availability

The raw data underlying this study are available via Zenodo at 10.5281/zenodo.19474316 (ref. ^39^).
