# Supplementary material for: Evaluating perfusion and metabolic responses of microvascular free flaps to ischemia, reperfusion and fluid resuscitation during septic shock in a large animal model
Source: Lab Anim (NY). 2026 May 20;55(8):314–26. doi: 10.1038/s41684-026-01747-0 (PMC13421353; doi:10.1038/s41684-026-01747-0)
Supplement: Supplementary file 1 — Supplementary Figs. 1 and 2 and Table 1. [file 41684_2026_1747_MOESM1_ESM.pdf]

lab animal

https://doi.org/10.1038/s41684-026-01747-0

Evaluating perfusion and metabolic responses of microvascular free flaps to ischemia, reperfusion and fluid resuscitation during septic shock in a large animal model

In the format provided by the authors and unedited

Supplementary Material

Surgical Procedure

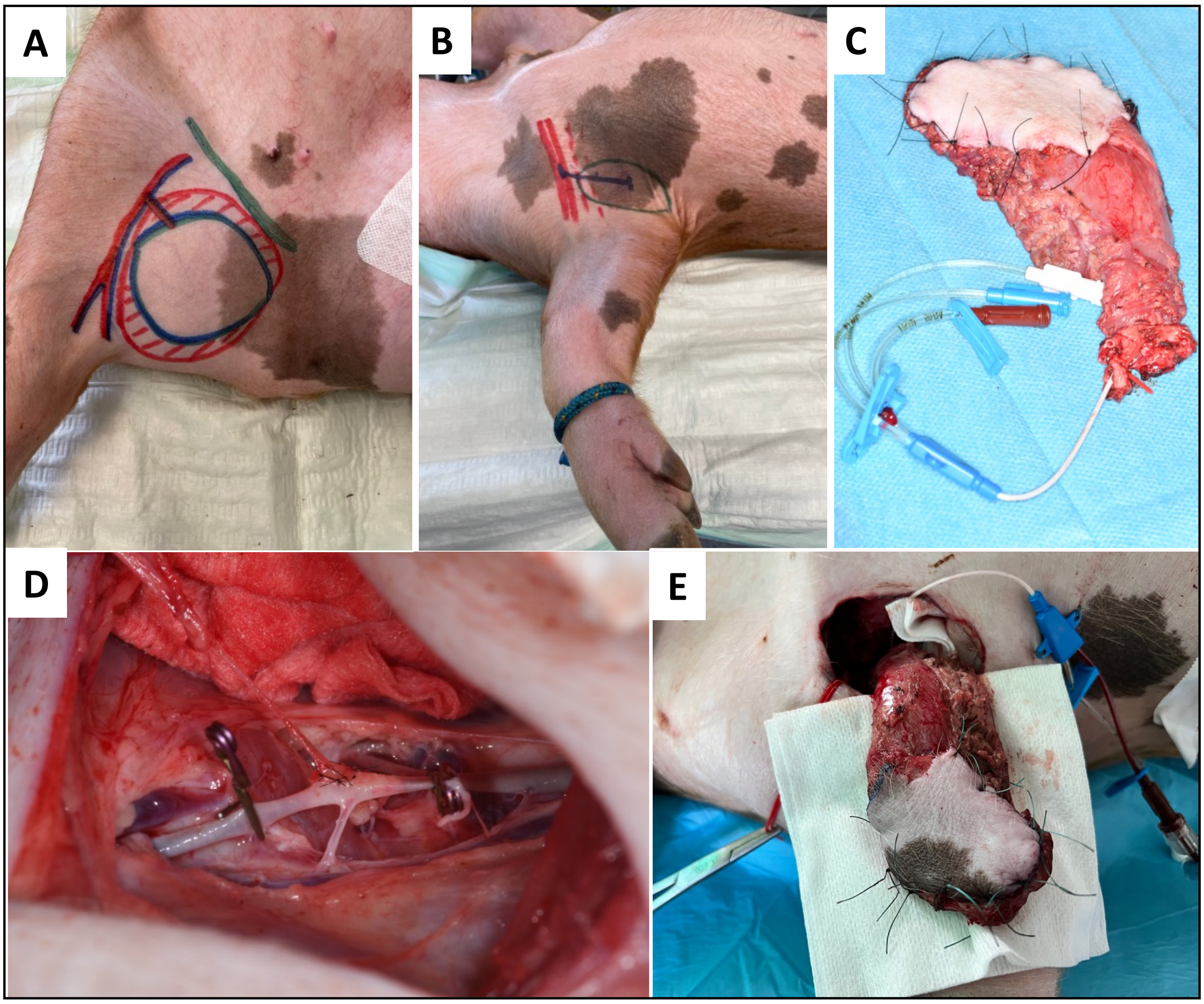

Figure 1S: Anesthetized and intubated pig in supine position with abducted legs: Anatomical relevant structures were drawn in projection on the skin using color markers, including the inguinal ligament (green), the gracilis muscle (red-hatched), the skin paddle (blue ellipse), and the flaps' vascular pedicle (A). Schematic marks further indicate the axilla (green), pectoralis muscle (red), and incision course (blue) (B) for dissection and skeletonization of the axillary artery. Following elevation of the gracilis myocutaneous flap, the venous pedicle was catheterized using a venous catheter system with an elastic rubber fixation (C). The flap was transferred to the axillary recipient site, and an end-to-side microvascular anastomosis was performed to re-establish circulation (D). After completion of the surgery, the reperfused flap was positioned in the axilla, with the venous catheter in place allowing repeated intra-flap blood sampling (E).

Table 1S: Comprehensive overview of measurement timepoints

T1	Start of the experiment, baseline data before any intervention
T2	Intra-surgery: after flap harvest but before vessel dissection
T3	Intra-surgery: after flap transfer and arterial microanastomosis, immediately before vessel opening and reperfusion
T4	Intra-surgery: immediately after flap perfusion
T5	End of shock phase and start of fluid therapy
T6	Circulatory stabilization
T7	1h hour after stabilization (observation period)
T8	2h hour after stabilization (observation period)
T9	3h hour after stabilization (observation period)

Experimental Setup

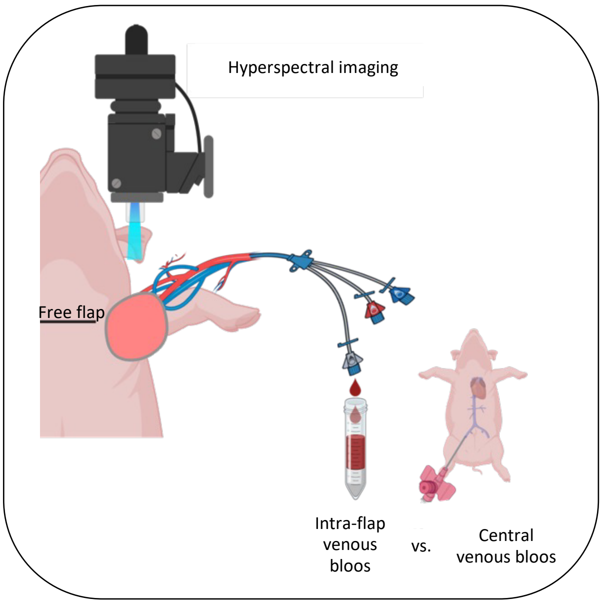

Figure 2S: Schematic illustration of the experimental setup, depicting the myocutaneus gracilis flap with its catheterized venous pedicle for direct intra-flap blood withdrawal, the parallel collection of central venous blood, and the positioning of the hyperspectral imaging system for non-invasive assessment of flap perfusion and tissue oxygenation. Created in BioRender. Thiem, D. (2026)
