# Supplementary material for: Evaluating perfusion and metabolic responses of microvascular free flaps to ischemia, reperfusion and fluid resuscitation during septic shock in a large animal model
Source: Lab Anim (NY). 2026 May 20;55(8):314–26. doi: 10.1038/s41684-026-01747-0 (PMC13421353; doi:10.1038/s41684-026-01747-0)
Supplement: Supplementary file 2 — Reporting Summary [file 41684_2026_1747_MOESM2_ESM.pdf]

nature portfolio

Corresponding author(s): Dr. Dr. Daniel Stephan

Last updated by author(s): Dr. Dr. Daniel Stephan (06.04.2026)

Reporting Summary

Nature Portfolio wishes to improve the reproducibility of the work that we publish. This form provides structure for consistency and transparency in reporting. For further information on Nature Portfolio policies, see our Editorial Policies and the Editorial Policy Checklist.

Please do not complete any field with "not applicable" or n/a. Refer to the help text for what text to use if an item is not relevant to your study.
For final submission: please carefully check your responses for accuracy; you will not be able to make changes later.

Statistics

For all statistical analyses, confirm that the following items are present in the figure legend, table legend, main text, or Methods section.

n/a	Confirmed	
☐	☒	The exact sample size (n) for each experimental group/condition, given as a discrete number and unit of measurement
☐	☒	A statement on whether measurements were taken from distinct samples or whether the same sample was measured repeatedly
☐	☒	The statistical test(s) used AND whether they are one- or two-sided<br>*Only common tests should be described solely by name; describe more complex techniques in the Methods section.*
☐	☒	A description of all covariates tested
☐	☒	A description of any assumptions or corrections, such as tests of normality and adjustment for multiple comparisons
☐	☒	A full description of the statistical parameters including central tendency (e.g. means) or other basic estimates (e.g. regression coefficient) AND variation (e.g. standard deviation) or associated estimates of uncertainty (e.g. confidence intervals)
☐	☒	For null hypothesis testing, the test statistic (e.g. F, t, r) with confidence intervals, effect sizes, degrees of freedom and P value noted<br>*Give P values as exact values whenever suitable.*
☒	☐	For Bayesian analysis, information on the choice of priors and Markov chain Monte Carlo settings
☐	☒	For hierarchical and complex designs, identification of the appropriate level for tests and full reporting of outcomes
☐	☒	Estimates of effect sizes (e.g. Cohen's d, Pearson's r), indicating how they were calculated

Our web collection on statistics for biologists contains articles on many of the points above.

Software and code

Policy information about availability of computer code

Data collection	Hyperspectral imaging data were collected using the TIVITA™ Tissue System (Diaspective Vision GmbH, Pepelow, Germany), and blood gas analyses were performed using the ABL 90 Flex blood gas analyser (Radiometer GmbH, Krefeld, Germany).
Data analysis	Statistical analyses were performed using GraphPad Prism 9.0 (GraphPad Software, LLC, Boston, USA), Excel 16.76 (Microsoft Corporation, Redmond, USA), and SPSS Statistics 29 (IBM Deutschland GmbH, Böblingen, Germany). No custom code central to the study was used.

For manuscripts utilizing custom algorithms or software that are central to the research but not yet described in published literature, software must be made available to editors and reviewers. We strongly encourage code deposition in a community repository (e.g. GitHub). See the Nature Portfolio guidelines for submitting code & software for further information.

Data

Policy information about availability of data

All manuscripts must include a data availability statement. This statement should provide the following information, where applicable:

- Accession codes, unique identifiers, or web links for publicly available datasets
- A description of any restrictions on data availability
- For clinical datasets or third party data, please ensure that the statement adheres to our policy

The raw data underlying this study have been deposited in Zenodo and are publicly available at https://doi.org/10.5281/zenodo.19474316

Research involving human participants, their data, or biological material

Policy information about studies with human participants or human data. See also policy information about sex, gender (identity/presentation), and sexual orientation and race, ethnicity and racism.

Reporting on sex and gender	This study did not involve human participants.
Reporting on race, ethnicity, or other socially relevant groupings	This study did not involve human participants.
Population characteristics	This study did not involve human participants.
Recruitment	This study did not involve human participants.
Ethics oversight	This study did not involve human participants.

Note that full information on the approval of the study protocol must also be provided in the manuscript.

Field-specific reporting

Please select the one below that is the best fit for your research. If you are not sure, read the appropriate sections before making your selection.

[X] Life sciences [ ] Behavioural & social sciences [ ] Ecological, evolutionary & environmental sciences

For a reference copy of the document with all sections, see nature.com/documents/nr-reporting-summary-flat.pdf

Life sciences study design

All studies must disclose on these points even when the disclosure is negative.

Sample size	Thirty-one juvenile male pigs (Sus scrofa domestica) were included in this prospective randomized trial. After shock induction, animals were allocated to three treatment groups: balanced crystalloid (n = 10), colloid (n = 11), and crystalloid plus resveratrol (n = 10).
Data exclusions	A total of 34 animals were initially prepared for the study. Three animals (nos. 12, 13 and 20) were excluded before the start of the experimental protocol because of cardiovascular instability and failure to reach the stage required for initiation of the experiment. These animals were therefore excluded prior to data collection and statistical analysis. The final analysed study population comprised 31 animals.
Replication	Measurements were obtained repeatedly within the same animals across predefined time points (T1–T9). Perfusion and metabolic parameters were assessed by repeated hyperspectral imaging and repeated blood gas analyses from intra-flap venous and central venous samples.
Randomization	Animals were randomized after shock induction to one of three resuscitation strategies using simple randomization by a lottery-draw system at the beginning of each experiment.
Blinding	Investigators were blinded to the assigned treatment group until the end of the surgical and shock-induction phase. After initiation of fluid resuscitation, blinding was no longer feasible because the fluids were visually distinguishable and required specific handling. Perfusion and metabolic parameters were obtained using automated and objective measurement systems.

Behavioural & social sciences study design

All studies must disclose on these points even when the disclosure is negative.

Study description	
Research sample	
Sampling strategy	
Data collection	
Timing	
Data exclusions	
Non-participation	
Randomization	

Ecological, evolutionary & environmental sciences study design

All studies must disclose on these points even when the disclosure is negative.

Study description

Research sample

Sampling strategy

Data collection

Timing and spatial scale

Data exclusions

Reproducibility

Randomization

Blinding

Did the study involve field work? ☐ Yes ☐ No

Field work, collection and transport

Field conditions

Location

Access & import/export

Disturbance

Reporting for specific materials, systems and methods

We require information from authors about some types of materials, experimental systems and methods used in many studies. Here, indicate whether each material, system or method listed is relevant to your study. If you are not sure if a list item applies to your research, read the appropriate section before selecting a response.

Materials & experimental systems

n/a	Involved in the study
☒	☐ Antibodies
☒	☐ Eukaryotic cell lines
☒	☐ Palaeontology and archaeology
☐	☒ Animals and other organisms
☒	☐ Clinical data
☒	☐ Dual use research of concern
☒	☐ Plants

Methods

n/a	Involved in the study
☒	☐ ChIP-seq
☒	☐ Flow cytometry
☒	☐ MRI-based neuroimaging

Antibodies

Antibodies used

Validation

Eukaryotic cell lines

Policy information about cell lines and Sex and Gender in Research

Cell line source(s)	
Authentication	
Mycoplasma contamination	
Commonly misidentified lines (See ICLAC register)	

Palaeontology and Archaeology

Specimen provenance	
Specimen deposition	
Dating methods	

☐ Tick this box to confirm that the raw and calibrated dates are available in the paper or in Supplementary Information.

Ethics oversight	

Note that full information on the approval of the study protocol must also be provided in the manuscript.

Animals and other research organisms

Policy information about studies involving animals; ARRIVE guidelines recommended for reporting animal research, and Sex and Gender in Research

Laboratory animals	Thirty-one juvenile male pigs (Sus scrofa domestica), 10–12 weeks old and weighing 31 ± 2.1 kg, were used. Animals were housed and handled in accordance with institutional and governmental regulations. Anaesthesia, instrumentation, surgical free flap transfer, and septic shock induction were performed under controlled laboratory conditions.
Wild animals	No wild animals were used.
Reporting on sex	Only male pigs were used.
Field-collected samples	No field-collected samples were used.
Ethics oversight	The study was approved by the State and Institutional Animal Care Committee Rhineland Palatine (approval no. G21-1-080) and complied with the German Animal Protection Law, the ARRIVE guidelines, and Directive 2010/63/EU.

Note that full information on the approval of the study protocol must also be provided in the manuscript.

Clinical data

Policy information about clinical studies

All manuscripts should comply with the ICMJE guidelines for publication of clinical research and a completed CONSORT checklist must be included with all submissions.

Clinical trial registration	This study did not involve a clinical trial.
Study protocol	This study did not involve human participants or clinical data.
Data collection	This study did not involve human participants or clinical data.
Outcomes	This study did not involve human participants or clinical data.

Dual use research of concern

Policy information about dual use research of concern

Hazards

Could the accidental, deliberate or reckless misuse of agents or technologies generated in the work, or the application of information presented in the manuscript, pose a threat to:

No | Yes
- [ ] | [X] Public health
- [ ] | [X] National security
- [ ] | [X] Crops and/or livestock
- [ ] | [X] Ecosystems
- [ ] | [X] Any other significant area

Experiments of concern

Does the work involve any of these experiments of concern:

No | Yes
- [X] | [ ] Demonstrate how to render a vaccine ineffective
- [X] | [ ] Confer resistance to therapeutically useful antibiotics or antiviral agents
- [X] | [ ] Enhance the virulence of a pathogen or render a nonpathogen virulent
- [X] | [ ] Increase transmissibility of a pathogen
- [X] | [ ] Alter the host range of a pathogen
- [X] | [ ] Enable evasion of diagnostic/detection modalities
- [X] | [ ] Enable the weaponization of a biological agent or toxin
- [X] | [ ] Any other potentially harmful combination of experiments and agents

Plants

Seed stocks

Novel plant genotypes

Authentication

ChIP-seq

Data deposition

- [ ] Confirm that both raw and final processed data have been deposited in a public database such as GEO.
- [ ] Confirm that you have deposited or provided access to graph files (e.g. BED files) for the called peaks.

Data access links
May remain private before publication.

Files in database submission

Genome browser session
(e.g. UCSC)

Methodology

Replicates

Sequencing depth

Antibodies

Peak calling parameters

Data quality

Software

Flow Cytometry

Plots

Confirm that:

- ☐ The axis labels state the marker and fluorochrome used (e.g. CD4-FITC).
- ☐ The axis scales are clearly visible. Include numbers along axes only for bottom left plot of group (a 'group' is an analysis of identical markers).
- ☐ All plots are contour plots with outliers or pseudocolor plots.
- ☐ A numerical value for number of cells or percentage (with statistics) is provided.

Methodology

Sample preparation

Instrument

Software

Cell population abundance

Gating strategy

☐ Tick this box to confirm that a figure exemplifying the gating strategy is provided in the Supplementary Information.

Magnetic resonance imaging

Experimental design

Design type

Design specifications

Behavioral performance measures

Imaging type(s)

Field strength

Sequence & imaging parameters

Area of acquisition

Diffusion MRI ☐ Used ☐ Not used

Preprocessing

Preprocessing software

Normalization

Normalization template

Noise and artifact removal

Volume censoring

Statistical modeling & inference

Model type and settings

Effect(s) tested

Specify type of analysis: ☐ Whole brain ☐ ROI-based ☐ Both

Statistic type for inference

(See Eklund et al. 2016)

Correction

Models & analysis

n/a	Involved in the study
☐	☐ Functional and/or effective connectivity
☐	☐ Graph analysis
☐	☐ Multivariate modeling or predictive analysis

Functional and/or effective connectivity

Graph analysis

Multivariate modeling and predictive analysis

This checklist template is licensed under a Creative Commons Attribution 4.0 International License, which permits use, sharing, adaptation, distribution and reproduction in any medium or format, as long as you give appropriate credit to the original author(s) and the source, provide a link to the Creative Commons license, and indicate if changes were made. The images or other third party material in this article are included in the article's Creative Commons license, unless indicated otherwise in a credit line to the material. If material is not included in the article's Creative Commons license and your intended use is not permitted by statutory regulation or exceeds the permitted use, you will need to obtain permission directly from the copyright holder. To view a copy of this license, visit http://creativecommons.org/licenses/by/4.0/
